# The Role of Nitric Oxide in Doxorubicin-Induced Cardiotoxicity: Experimental Study

**DOI:** 10.4274/Tjh.2013.0013

**Published:** 2014-03-05

**Authors:** Ayşenur Bahadır, Nilgün Kurucu, Mine Kadıoğlu, Engin Yenilme

**Affiliations:** 1 Karadeniz Technical University, School of Medicine, Department of Pediatric Hematology, Trabzon, Turkey; 2 Ankara Oncology Hospital, Department of Pediatric Oncology, Ankara, Turkey; 3 Karadeniz Technical University, School of Medicine, Department of Pharmacology, Trabzon, Turkey; 4 Karadeniz Technical University, School of Medicine, Department of Histology, Trabzon, Turkey

**Keywords:** Doxorubicin, nitric oxide, Nitric oxide synthase inhibitors

## Abstract

**Objective:** We evaluated the myocardial damage in rats treated with doxorubicin (DOX) alone and in combination with nitric oxide synthase (NOS) inhibitors.

**Materials and Methods:** Twenty-four male Sprague Dawley rats (12 weeks old, weighing 262±18 g) were randomly assigned into 4 groups (n=6). Group I was the control group. In Group II, rats were treated with intraperitoneal (ip) injections of 3 mg/kg DOX once a week for 5 weeks. In Group III, rats received weekly ip injections of 30 mg/kg L-NAME (nonspecific NOS inhibitor) 30 min before DOX injections for 5 weeks. In Group IV, rats received weekly ip injections of 3 mg/kg L-NIL (inducible NOS inhibitor) 30 min before DOX injections for 5 weeks. Rats were weighed 2 times a week. At the end of 6 weeks, hearts were excised and then fixed for light and electron microscopy evaluation and tissue lipid peroxidation (malondialdehyde). Blood samples were also obtained for measuring plasma lipid peroxidation.

**Results:** Weight loss was observed in Group II, Group III, and Group IV. Weight loss was statistically significant in the DOX group. Findings of myocardial damage were significantly higher in animals treated with DOX only than in the control group. Histopathological findings of cardiotoxicity in rats treated with DOX in combination with L-NAME and L-NIL were not significantly different compared with the control group. The level of plasma malondialdehyde in the DOX group (9.3±3.4 µmol/L) was higher than those of all other groups.

**Conclusion:** Our results showed that DOX cardiotoxicity was significantly decreased when DOX was given with NO synthase inhibitors.

## Abstract (tr)

**Amaç: **Bu çalışmada sıçanlarda doksorubisinin (DOX) tek başına ve nitrik oksit sentaz (NOS) inhibitörleri ile birlikte kullanımının kalp kası üzerinde yarattığı hasar araştırıldı.

**Gereç ve Yöntemler:** Çalışmada 12 haftalık 24 adet erkek “Sprague Dawley” sıçanı (ortalama ağırlık 262±18 gr) rastgele dört gruba (n=6) dağıtıldı. Grup I kontrol grubu olarak ayırıldı. Grup II’ye haftada bir kez intraperitoneal (ip) 3 mg/kg, beş hafta DOX uygulandı. Grup III’e DOX enjeksiyonundan 30 dakika önce ip 30 mg/kg L-NAME (nonselektif NOS inhibitörü) 5 hafta uygulandı. Grup IV’e DOX enjeksiyonundan 30 dakika önce ip 3 mg/kg L-NIL (indüklenebilir NOS inhibitörü) 5 hafta uygulandı. Çalışma süresince sıçanlar haftada iki kez tartıldı. Altıncı haftada kalbin sol ventrikülünden alınan parçalardan elektron ve ışık mikroskobik inceleme ve lipit peroksidasyonu tayini (malondialdehid) yapıldı. Plazma lipit peroksidasyonunun belirlenmesi amacı ile sıçanlardan kan alındı.

**Bulgular:** Grup II, Grup III ve Grup IV’te kilo kaybı gözlendi. DOX uygulanan grupta istatistiksel olarak anlamlı kilo kaybı saptandı. Miyokard hasarı bulguları tek başına DOX alan hayvanlarda kontrol grubuna oranla daha yüksek oranda izlendi. DOX’le beraber L-NAME ve L-NIL verilen sıçanlarda kardiyotoksitenin histopatolojik bulguları kontrol grubundan farklı değildi. Plazma malondialdehid düzeyi DOX grubunda (9,3± 3,4), diğer tüm gruplara göre daha yüksek olarak ölçüldü.

**Sonuç:** Bizim çalışmamızda DOX’la beraber NOS inhibitörleri uygulandığında DOX’a bağlı kardiyak toksisite bulgularında anlamlı azalma olduğu gösterilmiştir.

## INTRODUCTION

Doxorubicin (DOX) is an anthracycline-group antibiotic that is commonly used in the treatment of childhood tumors, and 60% of the treatment protocols include anthracycline-group agents. The most significant side effect of these drugs cardiotoxicity [1,2]. Today, long-term side effects such as cardiac toxicity are more frequently observed due to the increased rates of survival in childhood cancers.

Cardiotoxicity secondary to anthracyclines is dependent on the cumulative dose. The patients may consequently experience irreversible chronic cardiomyopathy and congestive cardiac failure [1,3]. Several trials have been performed on the mechanism of DOX-associated cardiotoxicity. Currently the most widely accepted opinion is the injury of myocardial cells by free radicals occurring during DOX metabolism [1,4,5]. Trials have demonstrated that the risk of cardiotoxicity could be decreased by using free radical scavengers [6,7,8].

Nitric oxide (NO) is a potent vasodilator and a significant mediator in myocardial contraction, and it is shown to be involved in the pathogenesis of cardiac diseases such as cardiac failure, ischemia/perfusion injury, and cardiomyopathy [9]. Recent trials have demonstrated that NO is involved in the cardiotoxicity associated with DOX. It was shown that DOX increased NO synthesis in plasma and cardiac tissue [10,11]. NO increase is believed to be mediated by inducible NO synthase (iNOS) and endothelial NO synthase (eNOS) enzymes [12,13].

This trial was designed to investigate the role of NO in the cardiotoxicity induced by DOX in rats. For this purpose, the extend of the myocardial injury in the rat heart and the level of lipid peroxide products were assessed. 

## MATERIALS AND METHODS

Twenty-four 12-week-old male Sprague Dawley rats were used in the study. The animals were housed in cages with free access to food and water. The cages were placed in a quiet and temperature- and humidity-controlled room (22±2 °C and 60±5%, respectively) in which a 12:12-h light-dark cycle was maintained. The weight of the rats varied between 240 and 320 g (mean: 262±18 g). During the trial, the rats were evaluated for their health status twice daily and any changes in their activity were recorded. The rats were also weighed twice weekly. Experiments were conducted between 09:00 and 17:00 hours to minimize diurnal variation. The experimental protocol was approved by the Institutional Animal Ethics Committee of the Karadeniz Technical University Medical School. Animals were allowed a 1-week acclimatization period before being used in experiments.

The cardiac toxicity results obtained by the use of DOX alone (Carlo Erba, Turkey) and in combination with NOS inhibitors were evaluated. Among the NOS inhibitors, NG-nitro-L-arginine methyl ester (L-NAME, Sigma), a nonselective inhibitor, and N6-(1-iminoethyl)-L- lysine (L-NIL, Sigma), an inducible NO inhibitor, were used. 

The rats were randomized into 4 groups, each consisting of 6 rats. 

Group I: Control group. The rats in this group received saline injection at a dose of 10 mL/kg via intraperitoneal route once a week for 5 weeks.

Group II: DOX group. In this group, the rats received DOX injection at a dose of 3 mL/kg via intraperitoneal route once a week for 5 weeks (total cumulative dose: 15 mg/kg).

Group III: DOX + L-NAME group. Differently from the rats in the second experimental group, the rats in this group were also injected with L-NAME at a dose of 30 mg/kg via intraperitoneal route for 5 weeks 30 min before the injection of DOX once a week. 

Group IV: DOX + L-NIL group. Differently from the rats in the second experimental group, the rats in this group were also injected with L-NIL at a dose of 3 mg/kg via intraperitoneal route for 5 weeks 30 min before the injection of DOX once a week. 

**Surgical Excision of the Heart**

The animals were observed for 1 week after the last injection and the heart was surgically excised at the end of 6 weeks in the pharmacology department. Blood samples were drawn from the renal artery for determining the plasma lipid peroxidation products. The left ventricle of the excised heart was divided into 3 pieces and placed in the appropriate solutions for electron microscopic investigation, light microscopic examination, and lipid peroxidation assay.

**Light Microscopy and Electron Microscopy Study **

A portion of the excised left ventricle was placed in 10% formalin for light microscopy examination and another portion was placed in 2% glutaraldehyde fixative for electron microscopy examination. The preparations were examined and evaluated by a single histologist blind to the study using an Olympus BH2 light microscope and JEOL 1010 electron microscope, and pictures were subsequently taken.

The findings related to myocardial injury occurring secondarily to the toxic effect of DOX, including edema in the myocytes and interstitium, edema in the sarcoplasmic reticulum, vacuolization in the myocytes, loss of myofibrils, and injury of mitochondria (edema, atrophy, crista clustering, and loss of crista), were evaluated by using light and electron microscopy; the results were recorded either as absence (-) or presence (+) of injury. 

**Lipid Peroxide Assay**

For an indirect evaluation of the free radicals produced in each of the experimental groups, the experimental groups, lipid peroxidation products were measured in the rat heart and plasma. The level of malondialdehyde (MDA), a thiobarbiturate reagent, was determined via measurement in the biochemistry department.

**Statistical Method**

The statistical analyses were performed using SPSS for Windows. SPSS 13 (Statistical Package for Social Screnu) for Windows©” The results were expressed in terms of means±standard deviations and percentages. The toxicity results detected by electron microscopy and light microscopy in the DOX, DOX + L-NAME, and DOX + L-NIL groups were compared to the results from the control group. The DOX + L-NAME and DOX + L-NIL groups were also compared to each other.

The comparison of the numeric data (means) within the same group and for different groups was done using the Wilcoxon test followed by the Mann-Whitney U test for post-hoc analysis, while the nominal data (ratios) were compared using the Fisher test. Values of p≤0.05 were considered statistically significant. 

## RESULTS

During the 6-week monitoring period of the rats, 1 rat in the DOX + L-NIL group died after 2 weeks. The autopsy did not reveal the cause of death. During the monitoring period, the rats receiving DOX showed a marked reduction in their activity relative to the control group, while the rats in the DOX + L-NAME and DOX + L-NIL groups had better activity compared to the rats receiving only DOX. The rats did not exhibit any changes in health status except for the reduced activity.

The comparison of the weights revealed a 12% increase in rat weight from baseline to the end of the study in the control group (p=0.02), while the rats in Group II, Group III, and Group IV exhibited respective reductions in weight of 12%, 7%, and 6%. The highest weight reduction was detected in the group receiving DOX alone; there was a statistically significant difference between the initial and final weighing (p=0.02). The rates of weight loss were similar between Group III and Group IV and no statistically significant difference was detected between the initial and final weights of the rats in these groups (p=0.20 and p=0.78, respectively). 

**Figure 1:**
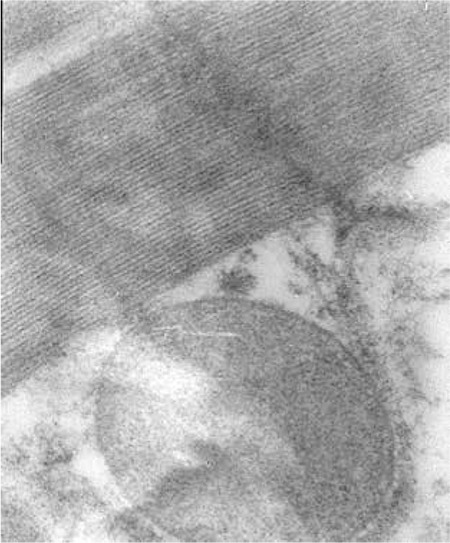
Group I=control; normal histopathological morphology, no findings of myocardial toxicity.

**Figure 2:**
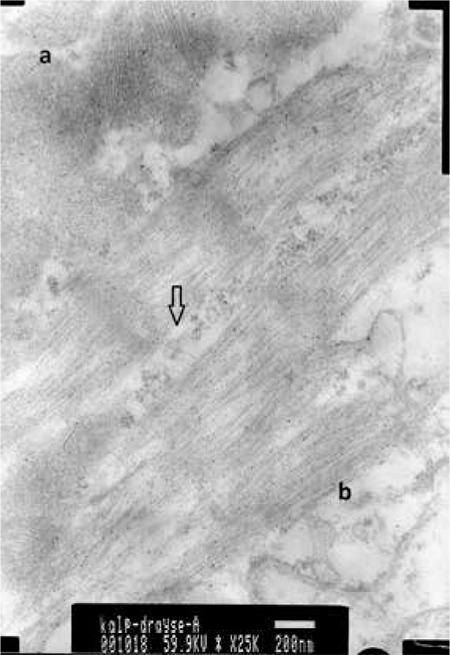
Group II =DOX; arrow shows the myocyte edema, ‘a’ shows mitochondrial injury, and ‘b’ shows sarcoplasmic reticulum edema. There is also loss of myofibrils.

**Figure 3:**
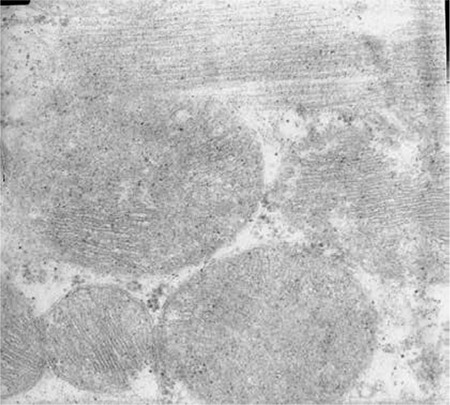
Group III=DOX + L-NAME; there is less mitochondrial injury.

**Figure 4:**
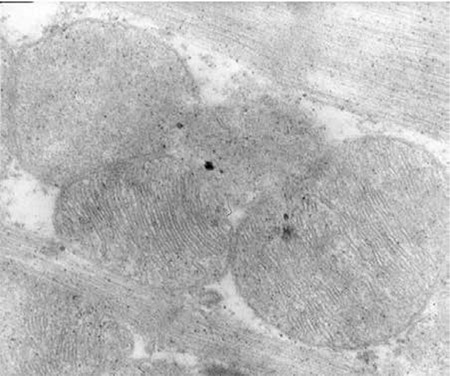
Group IV=DOX + L-NIL; less myocyte edema is observed.

The myocardial toxicities occurring with the use of DOX alone and in combination with the NOS inhibitors were assessed histopathologically by light and electron microscopy. Photos obtained in relation to the electron microscopic examination are presented in Figures 1, 2, 3, 4. 

While no findings of myocardial toxicity were detected in the rats in the control group, all the rats in Group II had at least one myocardial injury finding histopathologically. In 50% of the Group II rats, myocyte edema, vacuolization in myocytes, and loss of myofibrils were detected, while 83% had interstitial edema. Mitochondrial injury was detected in all rats.

In Group III and Group IV, myocardial toxicity was detected at a lower rate. None of the rats in Group III were detected to have myocyte edema, interstitial edema, vacuolization in the myocytes, or loss of fibrils, while 66% of the rats were observed to have sarcoplasmic reticulum edema and mitochondrial injury. As for Group IV, 80% of the rats did not have interstitial edema, sarcoplasmic reticulum edema, or vacuolization in the myocytes; 40% had myocyte edema and mitochondrial injury. There was no loss of fibrils (Table 1). 

The comparison of the histopathological cardiac toxicity findings (Table 2) revealed significantly increased rates of interstitial edema, sarcoplasmic reticulum edema, myocyte vacuolization, and mitochondrial injury in the group receiving DOX alone compared to the control group. There was no difference in the comparison of the myocyte edema and loss of myofibrils. In the group receiving L-NAME in combination with DOX, only the presence of sarcoplasmic reticulum edema was statistically significantly higher compared to the control group. There was no statistically significant difference in the toxicity findings of the group receiving L-NIL in combination with DOX when compared to the control group. 

With respect to the histopathological toxicity findings, the group receiving DOX alone had more marked findings; however, the difference from between the groups receiving L-NAME and L-NIL with DOX was not statistically significant. Binary comparison of the DOX + L-NAME and DOX + L-NIL groups revealed no statistically significant difference in the toxicity findings.

Upon comparison of the tissue and plasma MDA levels measured for demonstrating lipid peroxidation, the mean plasma MDA level (9.3±3.4 µmol/L) was detected to be higher in the DOX group compared to the other groups; the difference was statistically significant compared to the control group and the DOX + L-NAME group (p=0.03 for both). However, there was no marked difference between the groups with regard to the MDA values obtained from tissue. This was attributed to the difficulty of preparing homogenate from the cardiac tissue and the technical difficulty of the MDA assay.

## DISCUSSION

Several trials have been performed to determine the mechanisms involved in DOX-associated toxicity. The most widely accepted mechanism is the myocardial injury secondary to free radical formation. Anthracyclines were detected to increase the superoxide anion and hydrogen peroxide formation in the heart sarcosome, mitochondria, and cytoplasm depending on the drug concentration [1,5,6].

NO as the major regulator of the vascular tonus is significantly involved in cardiac functions and diseases. Cardiac NO production is mediated by the eNOS and iNOS enzymes [14,15,16]. While the basal NO production regulates the cardiomyocyte contractility and the blood flow, the excessively produced NO is involved in cardiac pathologies such as dilated cardiomyopathy and congestive cardiac failure [9,17,18,19]. Recent trials reported that NO was involved in the cardiotoxicity of DOX [10,11,13,20,21].

This trial was designed to investigate the role of NO in the DOX-associated cardiac toxicity. A higher rate of weight loss was detected in the rats receiving DOX alone in comparison to the rats receiving additional L-NIL and L-NAME. In addition, marked reduction in activity was detected. The literature data included reduced activity, weight loss, and acid formation as the clinical findings for DOX-associated toxicity. Guerra et al. reported that those with cardiomyopathy findings among rats that were administered a cumulative DOX dose of 13.5 mg/kg had significantly lower weight gain relative to the control group [11]. In the trial by Hirano et al., the rats receiving intravenous DOX weekly at a dose of 1.25 mg/kg (total dose: 5 mg/kg) and 2.5 mg/kg for 4 weeks (total dose: 10 mg/kg) exhibited reduction in weight in line with the cardiomyopathy findings; this was particularly marked in the group receiving the dose of 2.5 mg/kg/week [22].

The histopathological findings obtained in the cardiotoxicity models in empirical studies established by administering an intravenous or intraperitoneal dose of 1.5-3 mg/kg/week for 5 to 9 weeks (at cumulative doses of 10-20 mg/kg) were similar to findings in humans. The light and electron microscopy examination of rat heart resulted in reports of cytoplasmic vacuolization, myofibril loss, sarcoplasmic edema, and mitochondrial injury [4,23]. In our study, the histopathological investigation of the rat heart revealed marked findings of cardiac injury in the group receiving DOX. In particular, the interstitial edema (p=0.02), sarcoplasmic reticulum edema (p=0.002), vacuolization in the myocytes (p=0.02), and mitochondrial injury (p=0.002) were statistically significant compared to the control group. While toxicity findings were also detected in the groups receiving DOX + L-NAME and DOX + L-NIL, there was no statistically significant difference compared to the control group.

One of the first trials reported in the literature to investigate the contribution of NO to DOX-associated cardiac injury was performed by Guerra et al. Rats were administered 1.5 mg/kg of DOX for 9 weeks (total dose: 13.5 mg/kg) and the plasma NO levels were measured. The histopathological investigation of the rat hearts revealed findings of cardiac injury, and the plasma NO level was markedly higher compared to the control group. This study detected a positive correlation between the cardiomyopathy score and the NO level [11]. Sayed-Ahmed et al. measured the cardiac NO level after administering a single high dose (20 mg/kg) and gradually increasing daily doses of DOX (5-25 mg/kg) in rats. They reported that the cardiac NO levels were increased in cardiotoxicity induced with DOX at single and increasing doses. However, they were unable to detect any increase in the NO level and suggested that NO increase secondary to DOX was specific to the tissue [10]. In a trial where the culture of the rat cardiac cell was incubated with DOX for 24 h, DOX was reported to cause marked increase in NOS activity in the cells and the amount of NO in the supernatant, and this increase was inhibited by the addition of iron. This study concluded that DOX increased NO synthesis in the cardiomyocytes by affecting iron hemostasis [12]. However, we could not directly measure cardiac or plasma NO level due to lack of related laboratory systems.

The cardiac NO increase secondary to DOX was found to be mediated by iNOS. Aldieri et al. showed that the increase in the NO amount following treatment of the cardiac cells with DOX was associated with the increase in the iNOS gene expression [12]. Pacher et al. demonstrated that following DOX administration in mice with iNOS gene deletion, cardiac functions were better conserved [24]. The trial by Weinstein et al. demonstrated immunohistochemically that the myocardial iNOS was increased upon DOX administration [25]. Cardiac iNOS induction was shown to increase the myocardial injury secondary to oxidative stress by leading to the inactivation of the glutathione peroxidase, an intrinsic antioxidant [26]. 

After the contribution of myocardial NO formation to DOX cardiotoxicity was shown, the mechanism through which NO causes myocardial injury has been started to be investigated. The relevant trials have indicated that NO contributed to the peroxynitrite formation together with the superoxide formed by DOX. DOX toxicity is related to free radical formation. Oxygen and hydroxyl free radicals are produced via the DOX redox cycle catalyzed by flavor enzymes such as NADPH, cytochrome P450 reductase, and mitochondrial NADH dehydrogenase. These enzymes contribute to cardiomyopathy development secondary to DOX. NO is structurally similar to P450 reductase and was suggested to be involved in DOX metabolism [13,14,27].

All 3 isoforms of NOS have the capacity to catalyze the DOX redox cycle in the tumor tissue and form free radicals. DOX binds to the reductase domain of e-NOS and is reduced to the semiquinone form. Semiquinone causes formation of superoxide by reacting with the oxygen at a radical rate. All NOS isoforms form superoxide by reducing DOX under hypoxic conditions [13,14]. The high amount of NO produced by the iNOS enzyme causes formation of peroxynitrite by reacting with the superoxide anion. The resulting peroxynitrite oxides the cellular structures and contributes to myocardial oxidative injury, apoptosis, and necrosis by leading to lipid peroxidation [14,26,28,29].

Various studies demonstrated that NOS inhibitors prevented DOX cardiotoxicity. Administration of aminoguanidine, an iNOS inhibitor, with DOX to rats reduced the DOX mortality and acid formation and enhanced the histopathological changes in the rat heart [30]. Similarly, a trial by Pacher et al., performed on mice that were administered DOX in combination with aminoguanidine, showed a reduction in the cardiac dysfunction and mortality induced by DOX and improvement of the histopathological changes in the cardiac tissue [24]. Barnabe et al. added NOS inhibitors L-NAME and NG-monomethyl-L-arginine (L-NMMA) to medium before incubation with DOX in their study on cell cultures obtained from neonatal rat cardiac myocytes. They reported that pre-treatment administration of L-NAME and L-NMMA could prevent the myocyte injury associated with DOX [31]. In our study, we also observed reduction in the histopathological toxicity findings associated with DOX by L-NIL, a specific-selective iNOS inhibitor, and L-NAME, a nonspecific NOS inhibitor.

Packer et al. showed that MDA increased in the cardiac tissue as the lipid peroxidation product in mice that were administered DOX and were detected to have impairment in the left ventricle function; FP15, a peroxynitrite decomposition catalyst, prevented this increase [24]. In their trials, Fadılloğlu et al. administered an antioxidant, erdosteine, as a protective agent together with DOX, and they observed a marked increase in lipid peroxidation in the plasma and platelets in the group receiving DOX alone. Administration of erdosteine with DOX was shown to prevent intracellular and extracellular lipid peroxidation, thus exhibiting a protective effect. In addition, erdosteine was considered to inhibit the iNOS enzyme, thereby preventing NO production and thus peroxidation formation [32,33]. In our study, we also observed significantly increased MDA levels (a lipid peroxidation product) in rats receiving DOX relative to the control group. The L-NAME and L-NIL groups had a statistically significantly decreased MDA level compared to the DOX group. This represents a finding supporting the hypothesis that prevention of NO synthesis is protective against DOX cardiotoxicity.

## CONCLUSION

The assessment of both our results and the literature data suggest that NO, and especially that produced by iNOS, was involved in cardiac toxicity by leading to lipid peroxidation via peroxynitrite formation. An attempt to prevent NO production via inhibition of iNOS may be a solution for cardiac toxicity due to DOX in humans.

## CONFLICT OF INTEREST STATEMENT

The authors of this paper have no conflicts of interest, including specific financial interests, relationships, and/ or affiliations relevant to the subject matter or materials included.

## Figures and Tables

**Table 1:**
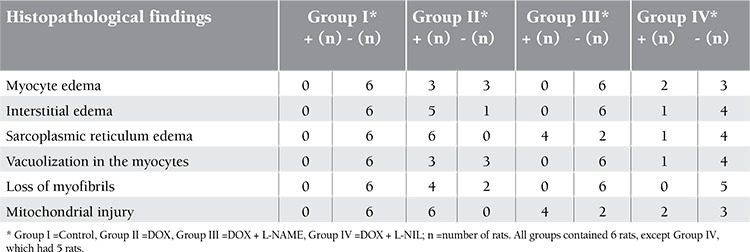
Histopathological evaluation of myocardial toxicity findings.

**Table 2:**
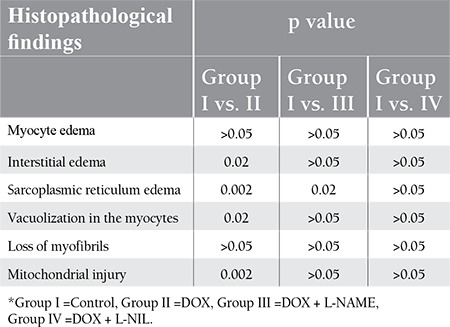
The comparison of the histopathological cardiac toxicity findings in the control group and drug groups.
